# Complete mitochondrial genome of the South American fur seal (*Arctocephalus australis*)

**DOI:** 10.1080/23802359.2017.1407692

**Published:** 2017-11-26

**Authors:** Pedro Rodrigues, Josefina Gutiérrez, Mauricio Seguel, Claudio Verdugo

**Affiliations:** a Instituto de Patología Animal, Facultad de Ciencias Veterinarias, Universidad Austral de Chile, Valdivia, Chile;; b Programa de Investigación Aplicada en Fauna Silvestre, Facultad de Ciencias Veterinarias, Universidad Austral de Chile, Valdivia, Chile;; c Department of Pathology, College of Veterinary Medicine, University of Georgia, Athens, GA, USA

**Keywords:** South America fur seal, Pinnipedia, mitogenome, Otariidae

## Abstract

The complete mitochondrial DNA sequence of the South America fur seal (*Arctocephalus australis*) was obtained by a shotgun sequencing approach. The mitogenome is 16,372 bp in length and includes the genes coding for the two rRNA species (12S and 16S), 13 protein-coding genes, 22 transfer RNA genes, and a control region. The base composition is 33.0% for A, 26.7% for C, 26.1 for T and 14.2% for G, with an overall GC content of 40.9%. The description of this mitogenome will be useful for further phylogeny and genetic studies on Pinnipeds.

The South American fur seal (*Arctocephalus australis*) is a Neotropical otariid species that occurs on the western South Atlantic and the eastern South Pacific coasts of South America (King 1983; Jefferson et al. 2015). Despite the intensive hunt for centuries, especially during the eighteenth and nineteenth centuries, leaving the species at the edge of extinction (Bonner 1982), the species is considered as Least Concern by the IUCN based on the thriving of the species in the last decades in the Atlantic Ocean (Cárdenas-Alayza et al. 2016). However, there are concerns on the conservation status of subpopulations in Peru and Southern Chile (Cárdenas-Alayza et al. 2016).

The use of genetic approaches is vital to understand the historical events that lead to the present distribution of species. The genomic mapping meaningfully increases the knowledge on species genome organization, allowing the genetic screen, identification, and location of major genes on populations (Crittenden et al. 1993), which could be used on the development of management and conservation measures, especially on species distributed along wide areas.

Total genomic DNA was extracted from liver tissue of five *A. australis* collected from necropsies performed at Isla de Guafo (43°35′ S, 74°42′ W), Chile, during Austral summer of 2016 and 2017, using a commercial kit (E.Z.N.A.^®^, Omega bio-tek, GA) and later sequenced on a MiSeq^®^, Illumina (San Diego, CA). The specimens were deposited on the Instituto de Patología Animal, Facultad de Ciencias Veterinarias, Universidad Austral de Chile (IPAFCV/N2/2016, IPAFCV/N6/2016, IPAFCV/N10/2016, IPAFCV/N4/2017, and IPAFCV/N5/2017). Raw reads were trimmed for Nextera DNA Library Preparation Kit (Illumina, CA) adaptors with Trimmomatic (Bolger et al. 2014) and by quality with Prinseq (Schmieder and Edwards 2011), and filtered for high-quality sequences using the public Galaxy server (http://galaxyproject.org). Reads were assembled using NextGENe^®^ software (Softgenetics^®^, State College, PA). A local BLAST search was used to select reads containing mitochondrial genome. The mitochondrial genomes of the *A. townsendi* (NC008420) and *A. forsteri* (KT693374) were downloaded and used to identify contigs containing mitochondrial sequences. The complete mitogenome was assembled and annotated in Geneious 9.1.2 (Kearse et al. 2012). CLUSTALW alignments against reference mitochondrial genomes were used to confirm manual annotations.

The mitochondrial genome of the *A. australis* (Accession MG023139) is 16,372 bp long, and includes 13 protein-coding genes, 22 transfer RNA genes, and a control region. The base composition of the mitogenome is 33.0% for A, 26.7% for C, 26.1 for T, and 14.2% for G, with an overall GC content of 40.9%. The overall mitogenome is 98% similar to *A. forsteri* and 95% to *A. townsendi*. The reconstructed phylogeny supported the placement of *A. australis* as sister to other *Arctocephalus clade* (Figure 1). Here, we report for the first time the complete mitochondrial genome of the *A. australis*, which could be important for further phylogenetic and population genetic studies on pinnipeds, especially of the genus *Arctocephalus*.

**Figure 1.**
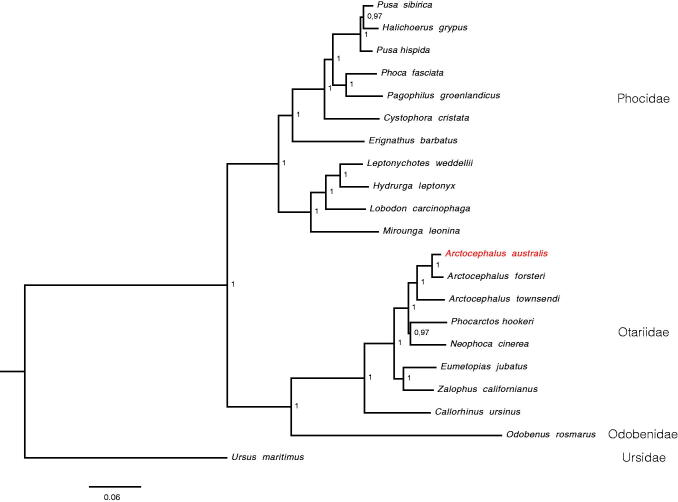
Bayesian inference tree based on 14,004 nucleotides of protein-coding mtDNA genes, reconstructed under GTR + I + G substitution model. Posterior probability values are indicated to each node.
